# Preparation of Multifunctional Dopamine-Coated Zerovalent Iron/Reduced Graphene Oxide for Targeted Phototheragnosis in Breast Cancer

**DOI:** 10.3390/nano10101957

**Published:** 2020-10-01

**Authors:** Chia-Hua Lin, Yi-Chun Chen, Pin-I. Huang

**Affiliations:** Department of Biotechnology, National Formosa University, Yunlin, 63208, Taiwan; a12345314@yahoo.com.tw (Y.-C.C.); superangela345@gmail.com (P.-I.H.)

**Keywords:** reduced graphene oxides, photodynamic therapy, photothermal therapy, nano zero-valent iron, magnetic resonance imaging

## Abstract

The present study aimed to develop a multifunctional nanoparticle platform with properties that are beneficial in imaging, targeting, and synergistic cancer phototherapy. To this end, we synthesized novel nanoparticles composed of polydopamine, nano zero-valent iron (nZVI), and reduced graphene oxide (rGO). We immobilized nZVI on the surface of GO (nZVI/GO), then further modified nZVI/GO with dopamine to form polydopamine-conjugated nZVI/rGO (nZVI/rGO@pDA). Because nZVI/rGO@pDA absorbs near infrared radiation (NIR) and binds biomolecules of cancer cells, this platform is highly efficacious in photothermal and photodynamic cancer therapy and enables specific targeting of breast cancer cells. Use of nZVI/rGO@pDA at a low concentration (10 μg/mL) resulted in irreversible damage to MCF-7 cells under NIR irradiation (808 nm) without inducing cytotoxic effects in normal cells. Furthermore, nZVI/rGO@pDA showed high sensitivity in magnetic resonance imaging (MRI), comparable to nZVI@pDA, even at low concentration. Monitoring the treatment response through evaluation of MRI signal intensity of nZVI/rGO@pDA in phototherapeutic therapy revealed that the novel material combines the advantages of nZVI, rGO, and pDA to provide specific targeting capabilities, excellent biocompatibility, and cancer phototherapeutic and tumor imaging abilities. Thus, this platform offers great potential in terms of imaging and therapeutic effects in phototherapy treatment for breast cancer.

## 1. Introduction

Phototherapy is a promising, noninvasive approach for the treatment of solid tumors [1,2]. The concept of phototherapy is based on two unique properties of photosensitizers: The generation of cytotoxic reactive oxygen species (photodynamic therapy, PDT) or the generation of heat (photothermal therapy, PTT), which are capable of killing cells through photoablation [3]. Overall, photosensitizers are considered harmless, as tumors can be treated precisely via selective irradiation, thus reducing the damage to surrounding healthy tissues [3]. However, most photosensitizers currently used for PDT require excitation by ultraviolet (UV) or visible light (Vis), thus limiting their deep-tissue penetration and therapeutic efficacy for tumor treatment. Furthermore, PDT has been shown to cause damage to tumor vasculature by direct effects on vascular endothelial cells [2]. Phototherapy represents a new promising technique for cancer therapy which uses nontoxic, light-sensitive compounds, with advantages over surgical methods and chemotherapy due to the ease of spatial/temporal control and minimal complications [1,4,5,6].

The near infrared (NIR) region of 700−1000 nm (the region of minimal light absorption for biological tissues) to allow efficient conversion of absorbed near-infrared optical energy into heat [7,8]. Recently, nanomaterial-based PTT agents have been widely investigated; including gold nanostructures, carbon nanomaterials (carbon nanotubes, graphene oxide [GO], and reduced graphene oxide [rGO]), and various other inorganic and organic nanomaterials with strong NIR absorbance; which can effectively convert the photo energy into heat to kill cancer cells under NIR irradiation [6,9,10,11,12,13,14]. Some studies have focused on multi-nanomaterials to achieve a combination of NIR-induced PTT and PDT into a single system through their enhanced therapeutic efficiency and minimal side effects relative to the individual therapeutic response [11]. However, most of these strategies are relatively complex and require multiple steps, and some require two different light sources to excite the PTT carrier and photosensitizers [15,16,17,18]; multi-laser treatment is very expensive and prolongs the required therapeutic time, which limits its clinical utility.

Both GO and rGO have been investigated for their potential application as photothermal therapy agents as they have high photothermal effects under low-power NIR irradiation due to their effective light-to-heat conversion compared with other carbon allotropes [4,19]. Several studies have employed GO or rGO as a vector to carry functional nanoparticles or specific nanocomposites with the aim of enhancing the therapeutic effects [20,21,22]. Nano zero-valent iron (nZVI) has been shown to have high catalytic activity in its catalysis of the decomposition of H_2_O_2_ to highly reactive •OH and •OOH via the Fenton and/or Fenton-like reactions [23,24,25,26]. When combined with UV irradiation, nZVI has high antibacterial potential due to the generation of reactive oxygen species (ROS) [27,28,29]. Therefore, nZVI/rGO nanocomposites represent ideal phototherapeutic agents for cancer therapy. However, the toxicity of nZVI and rGO in humans is a significant concern for the development of cancer drugs [30,31,32]. To increase the potential utility of nZVI/rGO-based nanomaterials in cancer therapy, dopamine has been used to modify nZVI and rGO to form a polydopamine (pDA) coating which exhibits excellent biocompatibility [19]. Dopamine shows quite strong optical absorption in the NIR range and is capable of effectively converting NIR light into heat [33,34,35,36]. In addition, dopamine receptors have been highlighted as therapeutic targets for breast cancer [37]. Therefore, combining dopamine and nZVI/rGO (nZVI/rGO@pDA) could lead to the creation of an effective phototherapeutic agent for breast tumors. Furthermore, nZVI can be transformed into iron oxide nanoparticles, which have magnetic properties after oxidation in the PDT process. Iron oxide nanoparticles are widely used in magnetic resonance imaging (MRI) as contrast agents due to their high biocompatibility and superparamagnetic properties [38,39,40]. Noninvasive MRI has been shown to be a powerful technique for high-resolution visualization of tumors [39].

In the present study, we synthesized a new multifunctional nanoparticle platform for targeted phototheragnosis of breast cancer tumors. This platform, namely, nZVI/rGO@pDA, was designed to enhance simultaneous MRI during phototherapy. Furthermore, we aimed to produce a novel material with therapeutic effects which would enhance imaging of tumors based on the phototherapeutic activity of dopamine, nZVI, and rGO and the enhancement of MRI imaging by nZVI-transformed iron oxide nanoparticles. We demonstrate that human MCF-7 breast cancer cells are targeted and killed by nZVI/rGO@pDA through ROS generation and temperature elevation. We performed MRI to supplement iron oxide nanoparticle-enhanced imaging and validate targeted therapy with nZVI/rGO@pDA as well as to carry out real-time monitoring of therapeutic efficacy. Our study shows that nZVI/rGO@pDA has anticancer activity in PDT/PTT, specific targeting capabilities, and enhances MRI imaging. These findings highlight the potential of this material as a potent phototheragnosis agent for breast cancer.

## 2. Materials and Methods 

### 2.1. Chemicals

Tris(hydroxymethyl)aminomethane (Tris), hydrochloric acid, boric acid, and all metal salts used in this study were purchased from Mallinckrodt Baker (Phillipsburg, NJ, USA). Potassium permanganate, sodium sulfide, and graphite (7–11 μm) were obtained from Alfa Aesar (Ward Hill, MA, USA). Hydrogen peroxide was purchased from SHOWA (Tokyo, Japan). Sulfuric and phosphoric acids were purchased from J. T. Baker (Phillipsburg, NJ, USA), and tris–hydrochloride was purchased from OmicsBio (Taipei, Taiwan). We purchased 3-(4,5-Dimethylthiazol-2-yl)-2,5-diphenyltetrazolium bromide (MTT), 2′,7′-dichlorodihydrofluorescein diacetate (DCFH-DA), dopamine hydrochloride and Prussian blue form Sigma-Aldrich (St. Louis, MO, USA). Phosphate-buffered saline (PBS) (0.01 M, pH 7.2) and fetal bovine serum were purchased form Gibco (Life Technologies, Thermo Fisher Scientific, MA, USA). Milli-Q ultrapure water (Millipore, Billerica, MA, USA) was used in all experiments. All chemicals were used without further purification.

### 2.2. Preparation and Characterization of nZVI/rGO@pDA

The synthesis routes for nZVI/rGO@pDA are shown in Scheme S1; GO was synthesized using an improved Hummers’ method [41] by adding a mixture of graphite flakes (1.5 g) and KMnO_4_ (9 g) to a mixture of concentrated H_2_SO_4_ and H_3_PO_4_. The mixture was then heated (50 °C) and stirred, then cooled to room temperature in an ice bath and poured into deionized water containing 30% H_2_O_2_. The aqueous mixture was centrifuged (35,000 g) for 1 h, and the resulting pellet repeatedly washed with deionized water until the wash solution reached pH 6.0. The aqueous solution was then sonicated and centrifuged. The GO solution was collected, and the remaining pellet discarded. 

We synthesized nZVI by mixing NaBH_4_ and FeCl_3_ solutions. The NaBH_4_ solution was titrated slowly into the FeCl_3_ solution to form nZVI [42]. We mixed nZVI with GO in a sodium phosphate solution (pH 7.4) and allowed to react for 1 h. The mixture was centrifuged (5000× *g*) to remove free nZVI. The supernatant was removed, and the precipitate washed with sodium phosphate solution. After washing for 3 cycles, nZVI/GO was resuspended in deionized water, then sonicated for 10 min and transferred to a round-bottomed flask. Tris-HCl and dopamine hydrochloride aqueous solution were injected into the flask under vigorous stirring. The synthesized nZVI/rGO@pDA was collected and washed with deionized water and, finally, suspended in deionized water and stored at 4 °C for no longer than 14 days. 

The optical properties and Raman spectra of nZVI/rGO@pDA were recorded using a UV-Vis spectrometer (Shimadzu, Kyoto, Japan) and LabRam-HR spectrometer (Jobin Yvon, France). The size, morphology, and thickness of nZVI/rGO@pDA were analyzed using AFM (Veeco, California, USA) and TEM (HT-7700, Hitachi High-Technologies Corporation, Tokyo, Japan). The quality of nZVI/rGO@pDA was evaluated by taking IS5 FTIR measurements in the range of 500–4000 cm^−1^ (Sigma, NO, USA). Zeta potential was analyzed using the Zetasizer 3000HS analyzer (Malvern Instruments, Malvern, UK).

### 2.3. Cell Cultures

Human breast cancer MCF-7 cells and human bronchial epithelium normal BEAS-2B cells were maintained in Dulbecco’s modified Eagle medium supplemented with 10% fetal bovine serum and LHC-9 medium, respectively, at 37 °C in a humidified atmosphere of 5% CO_2_ and 95% air. The culture medium was changed twice a week, and cells were passaged by trypsin every week.

### 2.4. Cytotoxic Potential of nZVI/rGO@pDA 

The MCF-7 and BEAS-2B cells were exposed to nZVI/rGO@pDA (0.5–10 μg/mL). Cell viability was determined using the MTT assay according to the manufacturer’s protocol using a spectrophotometer (Multilabel Reader, Perkin Elmer). Visible absorbance was recorded in a 96-well plate reader at 490 nm. Cell viability is expressed as the absorbance percentage relative to that of the control group.

### 2.5. Photothermal and Photodynamic Ability of nZVI/rGO@pDA

We irradiated nZVI/rGO@pDA suspensions in cell culture medium with an 808 nm NIR laser (PSU-H-LED, Taiwan). The temperature elevation and ROS formation were measured using thermocoupling and DCFH-DA assay. To examine the potential of nZVI/rGO@pDA in photothermal/photodynamic therapy, suspensions of nZVI/rGO@pDA (0.5–10 μg/mL) were added to the MCF-7 cells, which had been seeded into 96-well plates (at a final concentration of 8000 cells/well). Samples were irradiated with NIR at a power density of 1.5 W/cm^2^ for 3.5 min. The potential of nZVI/rGO@pDA in PTT/PDT was then assessed by thermocoupling and DCFH-DA assay, respectively. The DCFH-DA assay was used to determine ROS formation in cells after exposure to nZVI/rGO@pDA under NIR illumination. Briefly, MCF-7 cells were seeded in 96-well plates for 12–16 h. The media was then discarded from the plates and replaced with Dulbecco’s modified Eagle medium containing DCFH-DA and medium, GO, rGO@pDA and nZVI/rGO@pDA (10 μg/mL) under NIR illumination. ROS density was measured by fluorescence microscopy and Twinkle LB 970 fluorescence microplate reader in the darkness at 485 (excitation) and 535 nm (emission). H_2_O_2_ was used as a positive control (21-fold of control).

### 2.6. In Vitro Efficacy of PTT/PDT

We treated MCF-7 cells with nZVI/rGO@pDA (10 μg/mL) and/or NIR illumination (1.5 W/cm^2^, 3.5 min). Cell viability was measured using MTT assay immediately after irradiation, or after irradiation and 24 h of incubation with nZVI/rGO@pDA. Absorbance in the visible range was recorded in a 96-well plate reader at 490 nm. The PTT/PDT efficacy is expressed as absorbance percentage relative to the control.

### 2.7. Targeting Ability of nZVI/rGO@pDA

To examine the targeting ability of nZVI/rGO@pDA, cell culture medium containing nZVI/rGO@pDA (10 μg/mL) was added into the MCF-7 and BEAS-2B cell cultures and the cells incubated for 24 h. After this, cells were rinsed three times with PBS to remove any free nZVI/rGO@pDA. Cells were imaged to evaluated nZVI/rGO@pDA targeting using a light microscope.

### 2.8. Magnetic Resonance Imaging Ability of nZVI/rGO@pDA

To examine the MRI imaging ability of nZVI/rGO@pDA, solutions containing nZVI/rGO@pDA (0–100 μg/mL) were imaged in a 24-well plate on a 1.5-T MR system (Symphony, Siemens, Germany). The intensity of T_2_-weighted images was measured. For Prussian blue staining, the cell culture medium containing nZVI/rGO@pDA (10 μg/mL) was added into the MCF-7 cell culture for 24 h, then cells were rinsed three times with PBS to remove any free nZVI/rGO@pDA. The nZVI/rGO@pDA-treated MCF-7 cells were either irradiated with NIR or were not, then were incubated with potassium ferrocyanide in hydrochloric acid to evaluate intracellular iron oxide. Images of iron oxide-containing cells were obtained using a light microscope.

### 2.9. Statistical Analysis 

All data were compared using a one-way analysis of variance followed by Dunnett’s multiple-comparison test. Significance was considered at *p* < 0.05.

## 3. Results and Discussion

### 3.1. Preparation of nZVI/rGO@pDA

**Figure 1:**
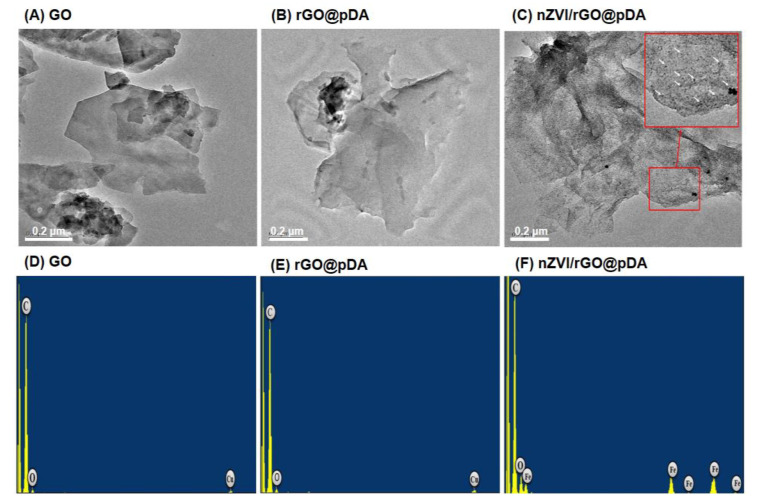
Transmission electron microscopy images of preparations. Images show representative micrographs of (**A**) GO, (**B**) rGO@pDA, and (**C**) nZVI/rGO@pDA. White arrows denote nZVI and the red boxes indicate higher magnification of the indicated area. The bottom panels show energy-dispersive X-ray spectra of (**D**) GO, (**E**) rGO@pDA, and (**F**) nZVI/rGO@pDA. Abbreviations: GO, graphene oxide; nZVI/GO, reduced graphene oxide modified with dopamine; nZVI/rGO@pDA, nano zero-valent iron immobilized on the surface of reduced graphene oxide then modified with dopamine.

**Figure 2:**
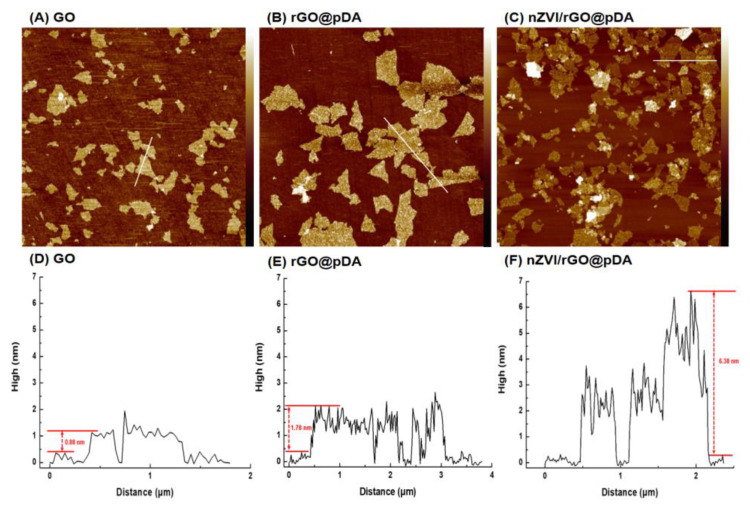
Representative tapping-mode atomic force microscopy images of preparations. Images show representative micrographs of (**A**) GO, (**B**) rGO@pDA, and (**C**) nZVI/rGO@pDA. Cross-section plots are also shown of (**D**) GO, (**E**) rGO@pDA, and (**F**) nZVI/rGO@pDA. Abbreviations: GO, graphene oxide; nZVI/GO, reduced graphene oxide modified with dopamine; nZVI/rGO@pDA, nano zero-valent iron immobilized on the surface of reduced graphene oxide then modified with dopamine.

Scheme S1 (see Scheme S1 in the Supporting Information) outlines the synthesis of nZVI/rGO@pDA. Both GO and nZVI were synthesized as previously reported [43]. The transmission electron microscopy (TEM) and atomic force microscopy (AFM) images showed that the GO was approximately 250 nm long and 0.88 nm thick (Figure 1A and Figure 2A,D). Mussel-inspired polydopamine was coated onto the GO surface to form 1.78 nm- and 6.38 nm-thick rGO@pDA and nZVI/rGO@pDA films (Figure 1B,C,E,F and Figure 2B,C,E,F). The TEM images of nZVI/rGO@pDA confirmed that nZVI were well distributed on the surface of GO and were approximately 5 nm in diameter (Figure 1C,F). 

**Figure 3:**
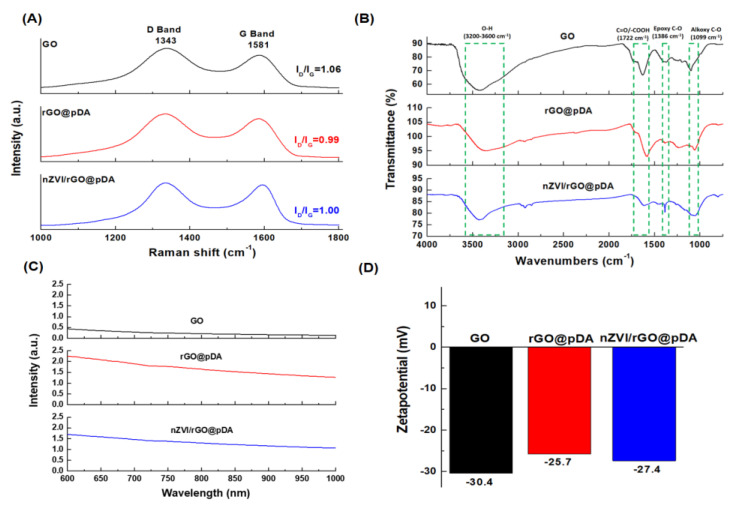
Physicochemical properties of nano zero-valent iron immobilized on the surface of reduced graphene oxide compared to modified with dopamine. (**A**) Raman spectra of prepared GO, rGO@pDA, and nZVI/rGO@pDA. (**B**) Fourier-transform infrared spectra of GO, rGO@pDA, and nZVI/rGO@pDA. (**C**) Ultraviolet-visible absorption spectra of GO, rGO@pDA, and nZVI/rGO@pDA. (**D**) Bar graph of zeta-potentials of GO, rGO@pDA, and nZVI/rGO@pDA. All experiments were performed three times independently. Abbreviations: GO, graphene oxide; nZVI/GO, reduced graphene oxide modified with dopamine; nZVI/rGO@pDA, nano zero-valent iron immobilized on the surface of reduced graphene oxide then modified with dopamine.

The Raman spectra of GO revealed the in-phase vibration of the graphene lattice (G band, sp^2^) to occur at 1581 cm^−1^, and the disorder band associated with graphene edges (D band, sp^3^) occurred at approximately 1343 cm^−1^ (Figure 3) [44]. When graphene is oxidized, functional groups are bonded to the surface and edges of graphite, resulting in some lattice defects. These defects eliminate the π–π resonance lattice mode. When graphene is oxidized, the degree of orderliness of the sp^2^ carbon structure gradually increases and the relative ID/IG intensity ratio was observed to increase. After pDA reduction, the ID/IG intensity ratio decreased from 1.06 to 0.99. When nZVI were coated onto the rGO surface, the ID/IG ratio increased to 1.00 (Figure 3A).

The various functional groups of GO, rGO@pDA, and nZVI/rGO@pDA were identified by Fourier-transform infrared (FTIR) spectroscopy. The FTIR spectrum for GO featured characteristic peaks for –OH groups (3200–3600 cm^−1^), C=O/–COOH groups (1722 cm^−1^), epoxy C–O groups (1386 cm^−1^), and alkoxy C–O groups (1099 cm^−1^) (Figure 3B) [45]. After modification with pDA, the intensity of peaks relating to the oxygen-containing functional groups decreased significantly in rGO@pDA and nZVI/rGO@pDA (Figure 3B) [19,46,47,48,49]. Furthermore, the absorption in the NIR region of rGO@pDA and nZVI/rGO@pDA were significantly enhanced, which may be attributable to the absorption of NIR light by pDA (Figure 3C). The zeta potentials of GO, rGO@pDA, and nZVI/rGO@pDA in the deionized water were −30.4, −25.7, and −27.4 mV (Figure 3D). Zeta potential is proportional to the force of electrostatic repulsion between particles; therefore, the high zeta potential of nZVI/rGO@pDA indicates that the nZVI/rGO@pDA-containing dispersion is highly stable [50].

### 3.2. Photothermal/Photodynamic Effects of nZVI/rGO@pDA

**Figure 4:**
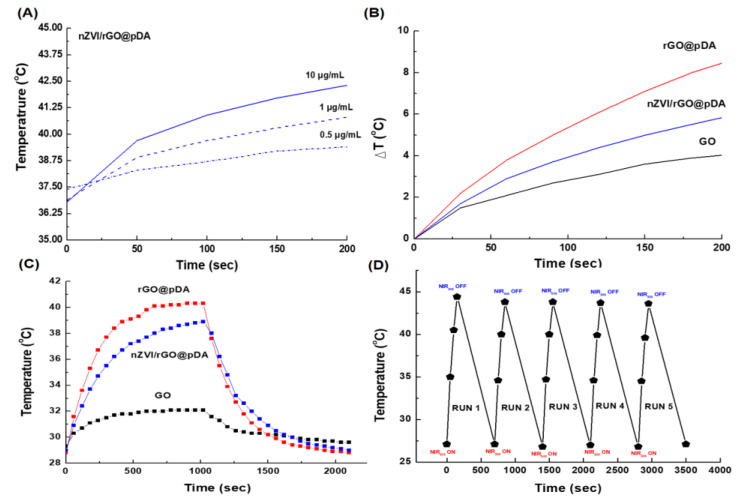
Photothermal effects of nano zero-valent iron immobilized on the surface of reduced graphene oxide compared to modified with dopamine. (**A**) Photothermal heating of different concentrations of nZVI/rGO@pDA (0.5–10 μg/mL) during NIR irradiation (880 nm, 1.5 W/cm^2^). (**B**) Photothermal heating of GO, rGO@pDA, and nZVI/rGO@pDA (10 μg/mL) during NIR irradiation (880 nm, 1.5 W/cm^2^). (**C**) Photothermal conversion efficiency of GO, rGO@pDA, and nZVI/rGO@pDA under NIR illumination. (**D**) Photothermal stability of nZVI/rGO@pDA under NIR illumination. All experiments were performed three times independently. Abbreviations: GO, graphene oxide; nZVI/GO, reduced graphene oxide modified with dopamine; nZVI/rGO@pDA, nano zero-valent iron immobilized on the surface of reduced graphene oxide then modified with dopamine.

Both rGO and pDA have been found to be effective photothermal tumor therapies under NIR irradiation [19,51,52]. After NIR irradiation, the temperatures of the nZVI/rGO@pDA solution increased with increasing irradiation time, and a concentration-dependent photothermal heating effect was observed, indicating that the temperature increased monotonically with nZVI/rGO@pDA (Figure 4A). After 2 min of NIR irradiation, the temperature of the 10 μg/mL nZVI/rGO@pDA solution was increased to 42.3 °C (Figure 4A). In comparison, the temperature of GO, rGO@pDA, and nZVI/rGO@pDA solutions (10 μg/mL) varied during NIR irradiation by +1.2 and +1.7 °C/min, respectively (Figure 4B). The temperature changes of the rGO@pDA solutions were larger, increasing by 2.5 °C/min (Figure 4B). In contrast, the cell culture medium, used as a control, exhibited only a slight temperature increase (0.18 °C/min) (data not shown). Both rGO@pDA and nZVI/rGO@pDA exhibited strong light absorption, making them ideal photothermal converters, and the rGO@pDA and nZVI/rGO@pDA with pDA and rGO preparations exhibited enhanced photothermal effect compared with GO alone [19,53]. The photothermal conversion efficiencies (η) of rGO@pDA and nZVI/rGO@pDA were calculated to be 29.6% and 24.1%, respectively, using the equation in SI1 (using the equation in S1 in the Supporting Information), which is much higher than that of GO (5.9%) (Figure 4C). In terms of photothermal stability, through five cycles of irradiation with NIR and cooling to room temperature, nZVI/rGO@pDA showed no significant variation during photothermal heating (Figure 4C), demonstrating the highly stable photothermal performance of nZVI/rGO@pDA.

**Figure 5:**
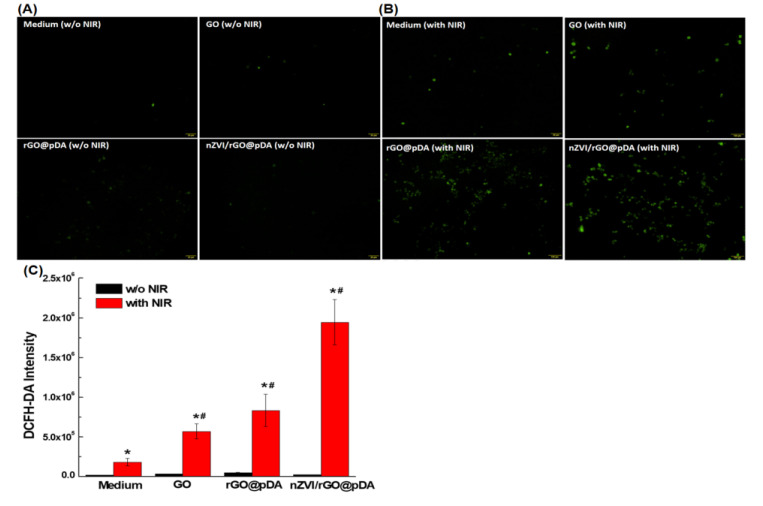
Photodynamic potential of nano zero-valent iron immobilized on the surface of reduced graphene oxide compared to modified with dopamine. Representative micrographs showing DCF fluorescence of MCF-7 cells after incubation with control solution, GO, rGO@pDA, nZVI/rGO@pDA (**A**) with and (**B**) without NIR irradiation. (**C**) Bar graph of quantitative analysis of reactive oxygen species formation (indicated by 2,7-dichlorofuorescin diacetate intensity) in MCF-7 cells following exposure to control solution, GO, rGO@pDA, or nZVI/rGO@pDA with/without NIR illumination. Key: * *p* < 0.05 compared with the control solution, GO, rGO@pDA, or nZVI/rGO@pDA without NIR illumination, *^#^ p* < 0.05 compared with the control solution with NIR illumination. All experiments were performed three times independently. Abbreviations: GO, graphene oxide; nZVI/GO, reduced graphene oxide modified with dopamine; nZVI/rGO@pDA, nano zero-valent iron immobilized on the surface of reduced graphene oxide then modified with dopamine.

We also monitored the level of intracellular ROS by evaluating the conversion of nonfluorescent 2,7-dichlorofuorescin diacetate (DCFH-DA) to fluorescent 2′,7′-dichlorofluorescein (DCF) in response to irradiation with NIR in cells which had been pre-cultured with GO, rGO@pDA, and nZVI/rGO@pDA. Widely used as a fluorescent probe, DCFH-DA is nonfluorescent but is oxidized to the highly fluorescent DCF by intracellular ROS [54]. After incubation with nanocomposites followed by DCFH-DA staining for 30 min at 37 °C, fluorescence spectrometry with excitation at 488 nm revealed the highest levels of ROS to exist in MCF-7 cells that had been incubated with nZVI/rGO@pDA nanocomposites (Figure 5). Compared with the blank medium, ROS accumulation following incubation with GO, rGO@pDA, and nZVI/rGO@pDA was increased by ~3.1-, 4.6-, and 10.6-fold (Figure 5).

### 3.3. Bio-Safety, Phototherapy Effect, and Targeting Ability of nZVI/rGO@pDA

**Figure 6:**
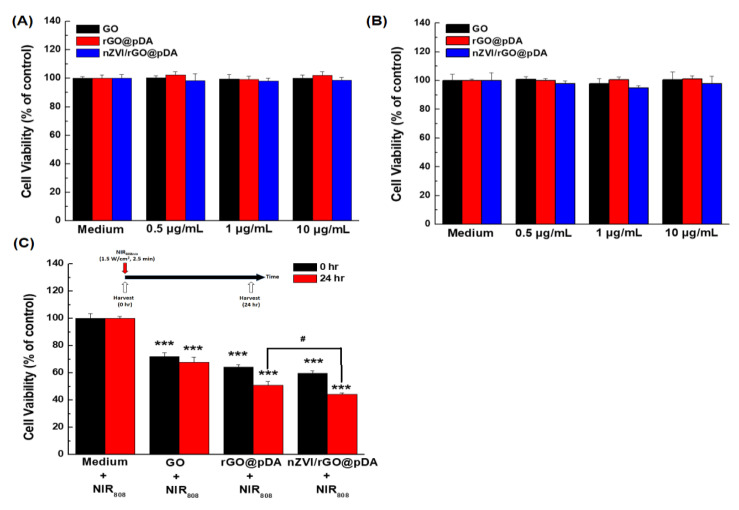
Bar graphs illustrating the biocompatibility and phototherapy effects of nano zero-valent iron immobilized on the surface of reduced graphene oxide then modified with dopamine. Bar graphs are shown illustrating: (**A**) Cell viability of MCF-7 cells after incubation with GO, rGO@pDA, nZVI/rGO@pDA; (**B**) cell viability of BEAS-2B cells incubated with GO, rGO@pDA, and nZVI/rGO@pDA; and (**C**) MCF-7 cell viability following exposure to GO, rGO@pDA, and nZVI/rGO@pDA under near infrared irradiation. Cell viability was measured by thiazolyl blue tetrazolium bromide assay. Key: *** *p* < 0.001 compared with the control, ^#^ *p* < 0.05 compared with cells exposed to rGO@pDA under near infrared illumination. All experiments were performed three times independently. Abbreviations: GO, graphene oxide; nZVI/GO, reduced graphene oxide modified with dopamine; nZVI/rGO@pDA, nano zero-valent iron immobilized on the surface of reduced graphene oxide then modified with dopamine.

The viability of both MCF-7 and BEAS-2B cells remained above ~95% after incubation with the nZVI/rGO@pDA, even at the highest concentration (10 μg/mL), for 24 h (Figure 6A,B). Our results suggest that nZVI/rGO@pDA has potential as a biocompatible material in PTT/PDT for cancer.

After demonstrating the bio-safety of nZVI/rGO@pDA, we performed NIR illumination experiments to evaluate the utility of nZVI/rGO@pDA for PTT/PDT of MCF-7 breast cancer cells. After treatment with GO, rGO@pDA, or nZVI/rGO@pDA (10 μg/mL) and NIR irradiation, the viability of MCF-7 cells was determined by thiazolyl blue tetrazolium bromide (MTT) assay. The results revealed that nZVI/rGO@pDA induced a more significant therapeutic effect than GO or rGO@pDA in this cell line (Figure 6C). To determine whether the therapeutic response was reversible, we compared the viability of MCF-7 cells incubated with nZVI/rGO@pDA for 24 h following NIR irradiation. As Figure 6C shows, the relative cell viability decreased continuously over time, suggesting that the phototherapeutic effect induced by nZVI/rGO@pDA leads to serious and irreversible damage to MCF-7 cells. 

**Figure 7:**
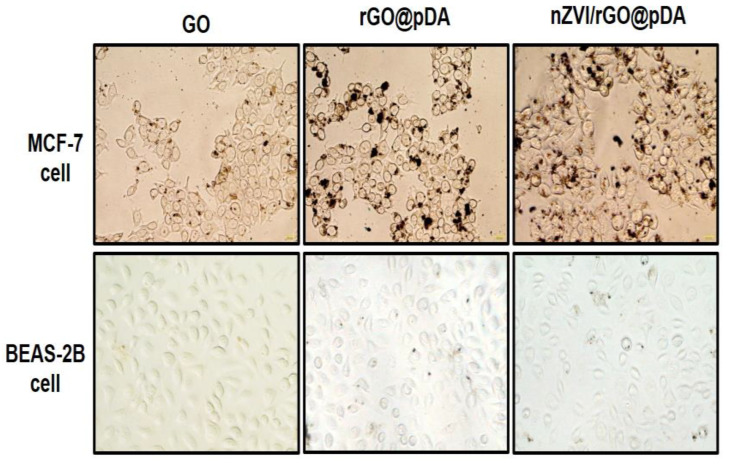
Targeting ability of nano zero-valent iron immobilized on the surface of reduced graphene oxide then modified with dopamine. Representative photomicrographs of MCF-7 and BEAS-2B cells incubated with GO, rGO@pDA, nZVI/rGO@pDA (10 μg/mL in the medium). All experiments were performed three times independently. Abbreviations: GO, graphene oxide; nZVI/GO, reduced graphene oxide modified with dopamine; nZVI/rGO@pDA, nano zero-valent iron immobilized on the surface of reduced graphene oxide then modified with dopamine.

Surface-immobilized pDA was used to specifically target breast cancer cells. As shown in Figure 7, treatment with rGO@pDA and nZVI/rGO@pDA for 24 h resulted in very few nanoparticles being deposited on BEAS-2B cells, while numerous rGO@pDA and nZVI/rGO@pDA were found to be bound to the surface of MCF-7 cells (Figure 7). These results demonstrate that rGO@pDA and nZVI/rGO@pDA can selectively target dopamine-receptor-positive MCF-7 cells and that nZVI/rGO@pDA has excellent tumor-targeting and phototherapeutic properties toward MCF-7 breast cancer cells. 

### 3.4. MRI Contrast Effect of nZVI/rGO@pDA

**Figure 8:**
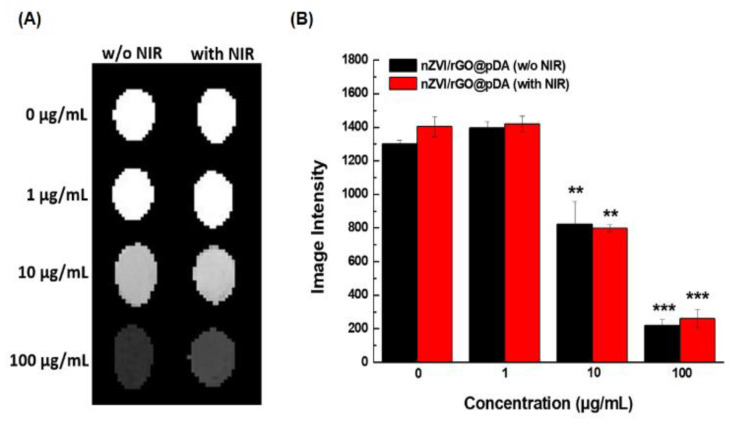
Analysis of magnetic resonance imaging contrast of nano zero-valent iron immobilized on the surface of reduced graphene oxide then modified with dopamine. (**A**) T_2_-weighted MRI of nZVI/rGO@pDA preparations (1–100 μg/mL) with/without near infrared NIR illumination. (**B**) Bar graph of the MRI signal intensities of nZVI/rGO@pDA (1–100 μg/mL) with/without NIR illumination. Key: ** *p* < 0.01 and *** *p* < 0.001 compared with the control. All experiments were performed three times independently. Abbreviations: GO, graphene oxide; nZVI/GO, reduced graphene oxide modified with dopamine; nZVI/rGO@pDA, nano zero-valent iron immobilized on the surface of reduced graphene oxide then modified with dopamine.

To evaluate the utility of nZVI/rGO@pDA as a contrast agent in MRI, the image intensity of different concentrations of nZVI/rGO@pDA suspensions (1–100 μg/mL) with/without NIR irradiation was measured. The image intensity of nZVI/rGO@pDA suspension increased in a dose-dependent manner (Figure 8), while the blank solution remained bright and indistinguishable (Figure 8). It is believed that MRI intensity is correlated with the amount of iron oxide internalized in a biological system [55]. Figure S1A–C confirms that nZVI/rGO@pDA was partially oxidized to iron oxide after exposure to NIR (see Figure S1 in the Supporting Information). Furthermore, the extent of nZVI/rGO@pDA oxidation increased over time (Figure S1D). It is likely that the MRI sensitivity of nZVI/rGO@pDA might also increase with increased oxidation of nZVI/rGO@pDA. These results indicate that the nZVI/rGO@pDA may be a useful nanomaterial for MRI-guided phototherapeutic treatment of breast cancers.

## 4. Conclusions

In the present study, we successfully developed multifunctional nZVI/rGO@pDA and demonstrated effective phototherapeutic inhibition of tumor MCF-7 cells, good breast-tumor-targeting ability, and sensitive detection by MRI. The nZVI/rGO@pDA that we prepared greatly facilitated breast tumor phototheragnosis at ultralow concentration, without toxic side-effects. This observation leads us to believe that nZVI/rGO@pDA has potential value in phototherapy and diagnostic imaging, representing a potential nanomedicine for future treatments of human breast cancer.

## Supplementary Materials

The following are available online at https://www.mdpi.com/2079-4991/10/10/1957/s1. Scheme S1 and Figure S1, which represent the preparation of nZVI/rGO@pDA nanocomposites and photomicrography of MCF-7 cells incubated with nZVI/rGO@pDA, and the photothermal conversion efficiency of nZVI/rGO@pDA.
