# Supplementary material for: Preparation of Multifunctional Dopamine-Coated Zerovalent Iron/Reduced Graphene Oxide for Targeted Phototheragnosis in Breast Cancer
Source: Nanomaterials (Basel). 2020 Oct 1;10(10):1957. doi: 10.3390/nano10101957 (PMC7601037; doi:10.3390/nano10101957)
Supplement: Supplementary file 1 [file nanomaterials-10-01957-s001.pdf]

Supporting Information

S1. Photothermal conversion efficiency of nZVI/rGO@pDA

The photothermal conversion efficiencies of nZVI/rGO@pDA were determined as previously described, with slight modification[1]. Changes in the temperature of nZVI/rGO@pDA suspensions were recorded as a function of time under continuous irradiation with NIR at 808 nm with a power intensity of 1.5 W/cm2. The temperature was also recorded real-time with data points taken every 10 s. The laser was shut off when the aqueous dispersion reached its highest temperature. To determine the rate of heat transfer, the temperature of the aqueous dispersion was continuously monitored. The photothermal conversion efficiency (η) was calculated using equation 1:

$$\eta = \frac{hS(T_{\max} - T_{surr}) - Q_{dis}}{I(1 - 10^{-A_{808}})}$$

where S is the surface area of the container; Tmax is the equilibrium temperature; Tsur is the surrounding temperature; Qdis represents the baseline energy input into the system, calculated by changing the aqueous solution of nZVI/rGO@pDA to pure water; I is the intensity of the laser input; and A808 is the absorbance of nZVI/rGO@pDA at 808 nm.

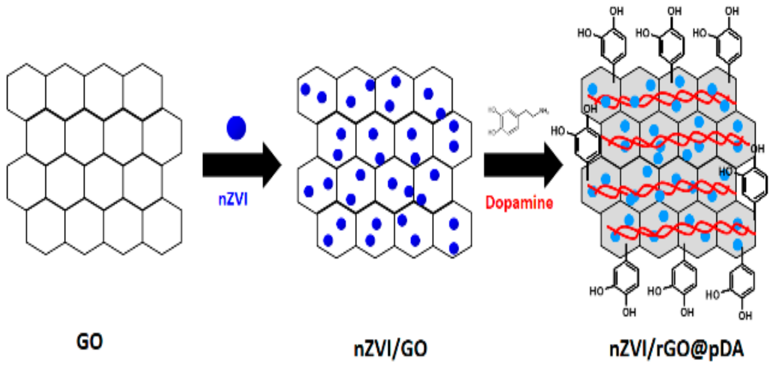

Scheme S1. Schematic representation of the preparation of nZVI/rGO@pDA nanocomposites.

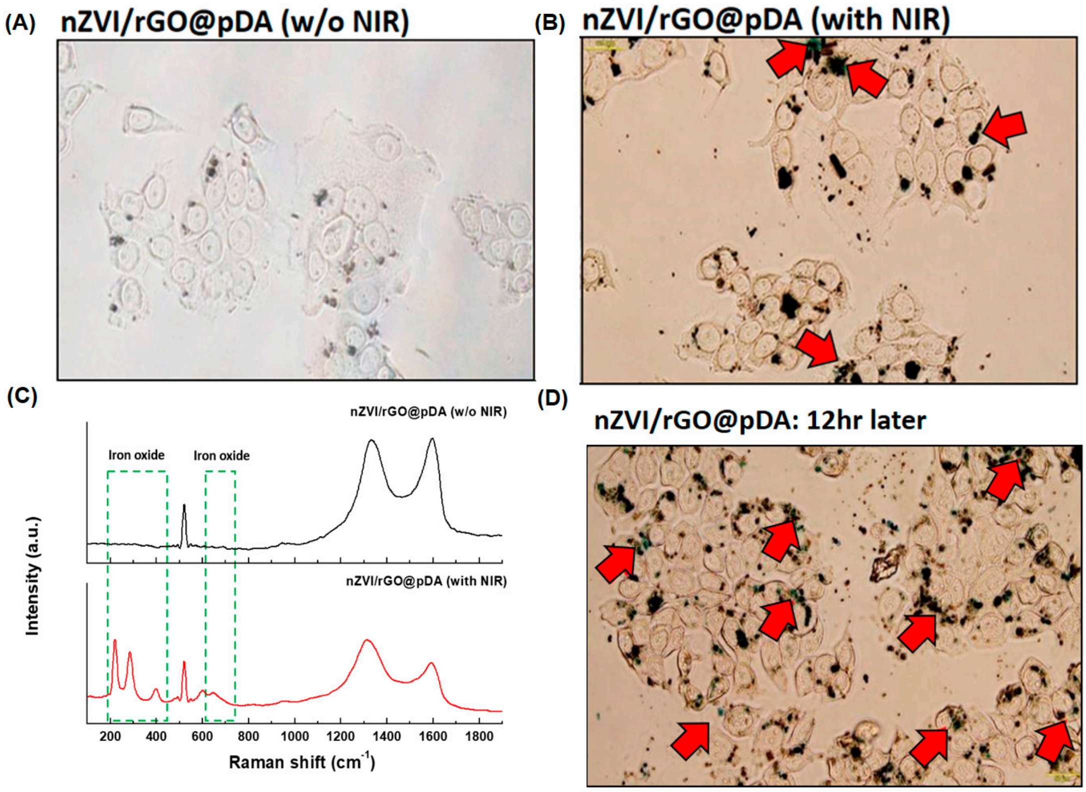

Figure S1 Photomicrography of MCF-7 cells incubated with nano zero-valent iron immobilized on the surface of reduced graphene oxide then modified with dopamine. Representative photomicrograph of MCF-7 cells incubated with nano zero-valent iron immobilized on the surface of reduced graphene oxide then modified with dopamine (nZVI/rGO@pDA) (A) without and (B) during near infrared (NIR) irradiation, and (D) after 12 h incubation and irradiation. Intracytoplasmic blue particles (red arrows in [B]) are clearly visible with Prussian blue staining. (C) Raman spectra of nZVI/rGO@pDA with/without NIR irradiation. Intracytoplasmic blue particles (red arrows in [D]) are clearly visible with Prussian blue staining. Abbreviations: a.u., absorbance units; GO, graphene oxide; NIR, near infrared; nZVI/GO, reduced graphene oxide modified with dopamine; nZVI/rGO@pDA, nano zero-valent iron immobilized on the surface of reduced graphene oxide then modified with dopamine.

References:

1. Roper, D.K.; Ahn, W.; Hoepfner, M. Microscale heat transfer transduced by surface plasmon resonant gold nanoparticles. *J Phys Chem C* **2007**, *111*, 3636-3641.
